# Extending Schelling’s segregation model from self orientation to social orientation

**DOI:** 10.1038/s41598-025-32159-8

**Published:** 2025-12-11

**Authors:** Unjong Yu, Kyuho Jin

**Affiliations:** 1 Department of Physics and Photon Science, Gwangju Institute of Science and Technology, Gwangju, 61005 South Korea; 2 School of Humanities and Social Sciences, Gwangju Institute of Science and Technology, Gwangju, 61005 South Korea

**Keywords:** Schelling’s segregation model, Microfoundation, Social orientation, Externality, Observability, Bounded rationality, Mathematics and computing, Physics

## Abstract

Schelling’s segregation model demonstrates how simple local rules can generate large-scale social patterns, yet it assumes agents act myopically and ignore the broader consequences of their moves. We extend this framework by introducing social orientation as a behavioral microfoundation, reflecting humans’ communal nature. Socially oriented agents follow boundedly rational heuristics that account for the satisfaction of prospective neighbors within a two-step horizon of observability, in addition to their own. We formalize this through three relocation rules—negative externality avoiding (NEA), positive externality favoring (PEF), and positive externality optimizing (PEO)—each capturing a different balance between minimizing disruption and promoting stability. Agent-based simulations reveal that these rules, while maintaining satisfaction, consistently reduce segregation, accelerate the attainment of global stability, and lower relocation moves per agent, thereby reducing social costs. These results provide a stronger behavioral foundation for segregation modeling and show how locally rational, socially sensitive decision-making can scale into equilibria that are not just welfare-enhancing but also yield more integrated and resilient communities.

## Introduction

Schelling’s segregation model, first introduced in the late 1960s through a checkerboard thought experiment^1,2^ (for background on a lesser-known yet significant earlier study, see Refs.^3,4^), remains one of the most striking illustrations of how simple micro-level rules can yield surprising macro-level patterns. With only minimal assumptions about agents’ preferences—namely, that they are content with some diversity but dissatisfied with being in a minority—Schelling showed that large-scale segregation can emerge even when no agent desires it^5,6^. This counterintuitive finding helped launch a broader research agenda on self-organization and unintended consequences, influencing not only economics and sociology but also the study of complexity and emergent order across the natural and social sciences^7,8^. Yet despite its enduring influence, the model’s stark simplicity leaves open important questions about the robustness of its insights under more realistic assumptions.

Subsequent work has examined the model’s generalizability by relaxing its assumptions, particularly those concerning agents’ microfoundations^9–13^. One microfoundation that has been relatively overlooked is that humans are inherently social beings. At their core, people are *homo sociologicus*^14,15^—defined by a fundamental inclination to form and belong to groups, with decisions shaped by communal considerations. Extensive empirical research supports this view: cooperation and reciprocity are evolutionarily adaptive^16,17^, identity and agency emerge through social interaction^18,19^, and social norms and roles perpetuate these orientations^20,21^. Together, these insights suggest that social orientation is an evolutionarily grounded and institutionally sustained behavioral tendency. From this perspective, a neighborhood is not merely a physical residence but also functions as a social community that builds and sustains mutually beneficial social capital through repeated interactions^22–29^. Accordingly, frequent turnover of neighbors signals the erosion of such capital, diminishing the neighborhood’s value as a social group and reducing it to little more than a residence^27,28,30–33^. For socially oriented agents, neighborhoods that risk this kind of degeneration after their move are correspondingly less attractive relocation options. Consequently, they avoid moves that generate negative externalities—destabilizing the community, particularly by prompting prospective neighbors to leave—even if such moves would satisfy them. Instead, they choose moves that generate positive externalities, thereby stabilizing the community and sustaining social capital.

By contrast, agents in Schelling’s original model are essentially *homo economicus* and abstract away from these dynamics^34,35^. They evaluate neighborhoods myopically, moving into any vacant site that meets their threshold in the moment, without regard for the destabilizing externalities of relocation^5,6^. In doing so, they treat satisfaction as purely individualistic and assume away interdependent neighborhood social dynamics—a stylized simplification that diverges from real-world decision-making. Recent studies have explored the use of (Pigouvian) taxes to redirect agents’ self-interested behavior toward mitigating negative externalities^36,37^. However, these models generally assume that externalities are exogenously regulated by a social planner who is well informed about the direction of each move’s externalities and, on that basis, able to align individual incentives with collective welfare, while agents themselves remain *homo economicus*, with intrinsic preferences largely insensitive to the social consequences of their actions.

To address this gap, we extend Schelling’s model by introducing a “social orientation” to its microfoundations, complementing the original focus on self-orientation. In our formulation, socially oriented agents seek both self-serving and community-preserving locations; they select sites where their move not only satisfies their own preferences but also minimizes dissatisfaction (and thus disruption) and/or enhances the satisfaction (and thus stability) of the prospective neighborhood. Following Schelling’s original definition, an agent is considered satisfied when the number of neighbors of the same type is at least equal to that of neighbors of a different type. In essence, these agents aim to reduce negative externalities and/or increase positive ones. This orientation reflects the valuation of neighborhoods not merely as locations, but as cohesive social communities that foster a shared identity^27,28,38^. We operationalize this behavior through three heuristic relocation rules based on the prospective neighbors’ satisfaction:

- **Negative externality avoiding rule (NEA)**: Agent relocates to a site that satisfies their threshold and does not dissatisfy any currently satisfied neighbors, choosing randomly among such sites.
- **Positive externality favoring rule (PEF)**: Agent relocates to a site that satisfies their threshold and generates non-negative externalities by increasing (or at least not reducing) the number of satisfied neighbors, choosing randomly among such sites.
- **Positive externality optimizing rule (PEO)**: Agent relocates to a site that satisfies their threshold, generates non-negative externalities by increasing (or at least not reducing) the number of satisfied neighbors and minimizes the number of unsatisfied neighbors, with ties broken at random.

All three rules begin with the agent’s own satisfaction—a self-oriented criterion—but extend it with an additional layer of social orientation, each emphasizing a distinct focus: *minimizing negative externalities* or *enhancing positive ones*. **NEA** focuses on avoiding negative externalities by preventing moves that would cause currently satisfied neighbors to become dissatisfied, while still permitting unsatisfied neighbors either to become satisfied or to relocate. This reflects the view that satisfied neighbors are key contributors to the cultivation and maintenance of social capital. **PEF**, in contrast, aims to increase—or at least not reduce—the total number of satisfied neighbors. The emphasis here is on *aggregate numbers*, so some switches between satisfied and unsatisfied neighbors may occur, provided the overall count of satisfied neighbors is maintained or improved. In contrast to the previous two rules, **PEO** maximizes positive externalities by considering both satisfied and unsatisfied neighbors: it is similar to PEF but adds the condition of minimizing the number of unsatisfied neighbors. In doing so, PEO can increase the number of satisfied neighbors, subject to minimizing the number of unsatisfied ones. Notably, these rules are not exhaustive but are intended to provide a parsimonious, heuristic framework that captures key variations in behavioral approaches to externalities and thus serves as a foundation for future extensions.

Deeper insight into these rules can be gained by viewing them through the lens of Pareto efficiency^39,40^, a widely used concept in economics. Among them, NEA is strictly Pareto-improving, as it satisfies the moving agent without making others worse off. By contrast, PEF and PEO relax this criterion by focusing on aggregate satisfaction, aligning more with a utilitarian logic than with strict Pareto improvement. Whereas NEA favors stability by preserving satisfied neighbors, PEF and PEO are expected to reduce turnover more proactively by converting unsatisfied neighbors into satisfied ones.

Implementing this microfoundation requires refining two implicit assumptions of the original model. The first concerns agents’ observability. In Schelling’s formulation, agents consider only their immediate neighbors, leaving the scope of perception irrelevant. By contrast, socially oriented agents must anticipate how their relocation affects not only themselves but also the satisfaction of prospective neighbors. This requires observing neighbors of neighbors—a form of two-step observability, or horizon of observability, which has empirical support in studies of social influence and network perception^41^. The second refinement concerns agents’ bounded rationality^42–45^. In the original model, agents resemble automata, following simple threshold rules with no capacity for foresight. In our extension, by contrast, agents more closely approximate rational decision makers in that they can evaluate prospective neighborhoods and select sites that optimize future social spillovers. Yet their rationality remains bounded by limited information and computational constraints: they cannot access global knowledge or compute system-wide optima^46,47^. Instead, they rely on local heuristics^48,49^—such as the relocation rules we propose—behaving as greedy searchers within their horizon of observability^50,51^.

These two behavioral refinements underpin social orientation by allowing agents to anticipate the communal consequences of their moves while remaining subject to realistic cognitive and informational limits. They also make the model more realistic and provide the foundation for our simulation analysis of how social orientation alters segregation dynamics. More broadly, modeling social orientation refines theoretical understanding and highlights implications for the stability and diversity of social systems.

In the following section, we outline how these rules are implemented in our extended Schelling model. Our aim is to examine how social orientation alters relocation behavior and, in turn, shapes dynamical outcomes including segregation. To this end, we adapt the classic model to incorporate agents who follow NEA, PEF, PEO, or Schelling’s original relocation rule. We then outline the modeling framework, agent decision rules, and simulation design used to compare these approaches.

## Methods

To evaluate the role of social orientation in segregation dynamics, we extend Schelling’s original agent-based model of residential mobility. As noted, our extension is based on two behavioral refinements–two-step observability and bounded rationality–allowing agents to anticipate potential neighborhood instability based on local information and avoid moves that could generate dissatisfaction among the prospective neighbors. We systemically assess how individual-level social orientation shapes macro-level outcomes of segregation, satisfaction, and convergence speed.

### Relocation rules

We consider three socially oriented relocation rules, with Schelling’s original rule¹ serving as a benchmark. The **NEA**, **PEF**, and **PEO** rules extend the model by incorporating agents’ socially oriented behavior to account for externalities. The stepwise procedures for each rule are summarized below:

### Negative externality avoiding (NEA) rule

- Step 1. Randomly select one dissatisfied agent.
- Step 2. Identify all vacant sites where (i) the agent would be satisfied if moved there—that is, under the counterfactual condition of occupancy—and (ii) the move would not cause any currently satisfied neighbors to become unsatisfied.
- Step 3. If more than one such site exists, randomly choose one from this set.
- Step 4. Relocate the agent to this site.

### Positive externality favoring (PEF) rule

- Step 1. Randomly select one dissatisfied agent.
- Step 2. Identify all vacant sites where (i) the agent would be satisfied if moved there—that is, under the counterfactual condition of occupancy—and (ii) the move would not reduce the number of currently satisfied neighbors.
- Step 3. If more than one such site exists, randomly choose one from this set.
- Step 4. Relocate the agent to this site.

### Positive externality optimizing (PEO) rule

- Step 1. Randomly select one dissatisfied agent.
- Step 2. Identify all vacant sites where (i) the agent would be satisfied if moved there—that is, under the counterfactual condition of occupancy—and (ii) the move would not reduce the number of currently satisfied neighbors.
- Step 3. Among these sites, choose the site that leaves the fewest neighbors unsatisfied after relocation.
- Step 4. If more than one such site exists, randomly choose one from this set.
- Step 5. Relocate the agent to this site.

**NEA** is primarily concerned with avoiding the turnover of currently satisfied neighbors, while giving little attention to unsatisfied neighbors. As a by-product, the rule may cause unsatisfied neighbors either to become satisfied or to relocate. In this sense, NEA aims to avoid the negative externalities associated with an agent’s relocation. **PEF**, by contrast, seeks to increase—or at least not decrease—the number of currently satisfied neighbors, without regard for the switching of unsatisfied neighbors so long as the overall number of satisfied neighbors increases in the prospective neighborhood. Accordingly, PEF is more concerned with generating positive externalities from an agent’s move. **PEO** integrates both perspectives by addressing positive and negative externalities simultaneously. Like PEF, it permits positive externalities by allowing unsatisfied neighbors to become satisfied, but it goes further by minimizing the number of unsatisfied neighbors rather than merely avoiding turnovers. Table 1 summarizes these distinctions.

**Table 1:** Comparison of social oriented rules.

Rule	Primary focus	Treatment of neighbors	Externality orientation
NEA	Avoid turnover of currently satisfied neighbors	Ignore unsatisfied neighbors; some may become satisfied or move out as a side effect	Minimize negative externalities (avoids new dissatisfaction)
PEF	Increase or at least not decrease *the number* of satisfied neighbors	Allow unsatisfied neighbors to switch to satisfied; unconcerned with turnover if satisfaction increases overall	Generate positive externalities (permits satisfaction gains)
PEO	Balance positive and negative externalities	Like PEF, permit unsatisfied → satisfied switches, but also minimize the number of unsatisfied neighbors	Address both: permit positive externalities while minimizing negative ones

NEA focuses narrowly on avoiding the turnover of satisfied neighbors, thereby minimizing negative externalities but disregarding unsatisfied ones. PEF, in contrast, prioritizes generating positive externalities by permitting unsatisfied neighbors to become satisfied, even if some turnover occurs. PEO integrates both perspectives: like PEF, it allows for positive spillovers, but it further minimizes the number of unsatisfied neighbors, making it the most balanced rule in terms of managing both positive and negative externalities.

It is worth noting that although the two-step observability in our model is farther-sighted than in the original, the three proposed rules still operate as local heuristics, since agents cannot anticipate consequences beyond their perceptual horizon. This property aligns with our goal of enhancing realism by modeling agents as boundedly rational actors who rely on such heuristics. While agents are assumed to identify all vacant sites, this reflects environmental observability (or an *externally visible search space*) rather than global cognitive access, as recognizing visible cues such as empty houses or lots requires minimal cognitive effort compared with evaluating their suitability for relocation^52,53^ (i.e., perceptual observability vs. cognitive boundedness). The decision process therefore remains locally informed and computationally bounded. Taken together, these rules allow us to explore a spectrum of socially oriented decision-making—from simple harm-avoidance to active community optimization. By doing so, we can systematically assess how these behavioral refinements impact macro-level outcomes.

Formally, when considering a move to site $\:i$, an agent with two-step observability computes for each neighbor $\:j$ the fraction of same-type neighbors as

$$\:{h}_{j}\left(i\right)=\frac{1}{\left|N\left(j\right)\right|}\sum\:_{k\in\:N\left(j\right)}1\left\{{x}_{j}\left(i\right)={x}_{k}\left(i\right)\right\},$$.

where $\:N\left(j\right)$ denotes the set of neighboring agents of $\:j$, and $\:{x}_{m}\left(i\right)\in\:\{-1,\:+1\}$ represents the occupant of site $\:m$ after the focal agent is hypothetically relocated to site $\:i$. The indicator function $\:1\{{x}_{j}\left(i\right)={x}_{k}\left(i\right)\}$ equals 1 if neighbor $\:j$ and their neighbor $\:k$ are of the same type following the relocation, and 0 otherwise. Accordingly, $\:{h}_{j}\left(i\right)$ captures the updated fraction of same-type neighbors for each $\:j$, incorporating the agent’s prospective move.

Following Schelling’s original specification, neighbor $\:j$ is considered satisfied if $\:{h}_{j}\left(i\right)\ge\:\tau\:$, where $\:\tau\:$ denotes the satisfaction threshold. Consistent with Schelling’s model, we set $\:\tau\:=0.5$, meaning that an agent is satisfied when the number of neighbors of the same type is at least equal to the number of those of a different type. This specification allows the relocating agent to anticipate whether its move would maintain or disrupt the satisfaction of nearby agents within its two-step observational range. For illustration, under NEA, agents randomly choose a site where they would be satisfied if moved there and the move would not cause any currently satisfied neighbor $\:j$ to become unsatisfied (i.e., would not result in $\:{h}_{j}\left(i\right)<0.5$).

This assumption aligns with Friedkin’s empirical observation of the “horizon of observability,” which suggests that an individual’s social vision is limited and typically extends only to indirect social ties^41^. Consequently, agents can consider the broader consequences of their relocation on local neighborhood stability.

### Simulation design

The model is implemented on a two-dimensional square lattice with periodic boundary conditions, so that agents at the edges are connected to those on the opposite side. Each cell can be occupied by a single agent or left vacant, and agents belong to one of two groups of equal size. A fixed vacancy ratio (20% unless otherwise noted) provides sufficient mobility for agents to relocate. At initialization, agents and vacancies are randomly assigned to sites on the grid. Time advances in asynchronous sequential updates, so that only one agent moves per step. The process continues until either (i) all agents are satisfied, or (ii) no further moves are possible because dissatisfied agents cannot find admissible sites. The resulting configuration, or steady state of the dynamics, is regarded as an equilibrium or a state of global stability.

### Outcome measures

We track three primary outcomes. First, segregation is measured using the standard adjacency-based segregation index^13^:

$$\:S=\frac{1}{2N}\sum\:_{i,j}{A}_{ij}{T}_{i}{T}_{j}$$

where $\:N$ is the number of agents, $\:A$ represents the adjacency matrix of the neighborhood structure, and $\:{T}_{i}\:$denotes the type of agent at site $\:i$ (+ 1 or − 1 for occupied sites, 0 for vacancies). Thus, the segregation index $\:S$ measures how similar neighboring agents are. It adds up every pair of neighboring agents, multiplies their types ($\:{T}_{i}{T}_{j}$)—which equals 1 if they are the same and − 1 if they are different—and then divides by twice the total number of agents. A higher value of $\:S$ indicates that agents tend to be surrounded by neighbors of the same type (greater segregation), whereas a lower value reflects a more mixed local composition. A value of $\:S=0$ corresponds to the expected outcome under a random configuration, in which same-type and different-type neighbor pairs occur in equal proportion and no systematic pattern of segregation or integration is present. The maximum value of S is $\:{\Delta\:}/2$ in a $\:{\Delta\:}$-regular network as $\:N\to\:\infty\:$ and the proportion of vacant site $\:V=0$. Notably, $\:S$ is closely related to the energy $\:E$ of the spin-glass model, satisfying the relation $\:S=-E/N$^54,55^. Second, overall satisfaction is measured as the proportion of agents who meet their threshold condition at the end of the simulation. Third, the convergence step is quantified as the number of relocation steps required to reach equilibrium, serving as a proxy for the speed of system stabilization.

### Computational implementation

The model was implemented in Python 3.12 using standard scientific libraries (NumPy, pandas, and matplotlib). Random number generation was controlled by fixed seeds for reproducibility. For each parameter configuration, we conducted 10,000 independent replications to account for stochastic variability.

## Results

Our simulations reveal that social orientation non-trivially alters the dynamics of Schelling’s segregation model. Under the original rule, the model reproduces Schelling’s classic finding: even moderate individual preferences lead to considerable segregation. By contrast, socially oriented relocation consistently reduces segregation and accelerates convergence to equilibrium (i.e., global stability) with fewer relocation steps, all while preserving full satisfaction. These results suggest that incorporating social orientation aligns individual-level decision-making with macro-level outcomes that enhance social integration, stability, and collective welfare. We present the results in three parts, focusing respectively on satisfaction outcomes, segregation outcomes, and equilibrium dynamics.

### Baseline parameters

Unless otherwise specified, all simulations were conducted on a 32 × 32 grid with periodic boundary conditions, Moore neighborhoods, a vacancy ratio of 0.2, and a satisfaction threshold of 0.5. The Moore neighborhood corresponds to Schelling’s original configuration, comprising the agent’s own location and its eight surrounding sites. Each reported value represents the mean of 10,000 independent replications, with standard errors shown where applicable. These baseline settings provide a consistent reference point for evaluating how socially oriented relocation rules shape segregation outcomes. Baseline results are summarized in Table 2.

**Table 2:** Baseline simulation results under socially oriented relocation rules.

Rule	Satisfaction (%)	Segregation index (S)	Convergence steps	Moves per agent
Schelling	100.0 (0.0)	2.384 (0.001)	7.759 (0.017)	0.558 (0.001)
NEA	100.0 (0.0)	2.191 (0.001)	5.684 (0.011)	0.490 (0.001)
PEF	100.0 (0.0)	2.187 (0.001)	5.797 (0.011)	0.497 (0.001)
PEO	100.0 (0.0)	2.079 (0.001)	5.380 (0.011)	0.468 (0.000)

Simulations were conducted on a 32 × 32 grid with periodic boundary conditions, Moore neighborhoods, a vacancy ratio of 0.2, and a satisfaction threshold of 0.5. Each entry represents the mean of 10,000 replications, with standard errors in parentheses. Socially oriented relocation yields lower segregation, full satisfaction, and faster convergence, reducing both equilibrium time and moves per agent.

### Satisfaction outcomes

We first examine overall satisfaction levels across the three relocation rules. In all simulations, the system converges to complete satisfaction (a final satisfaction value of 1). This indicates that the rules do not impede the attainment of full satisfaction. Accordingly, our subsequent analysis focuses on segregation outcomes under a *ceteris paribus* condition with respect to satisfaction.

### Segregation outcomes

**Fig. 1:**
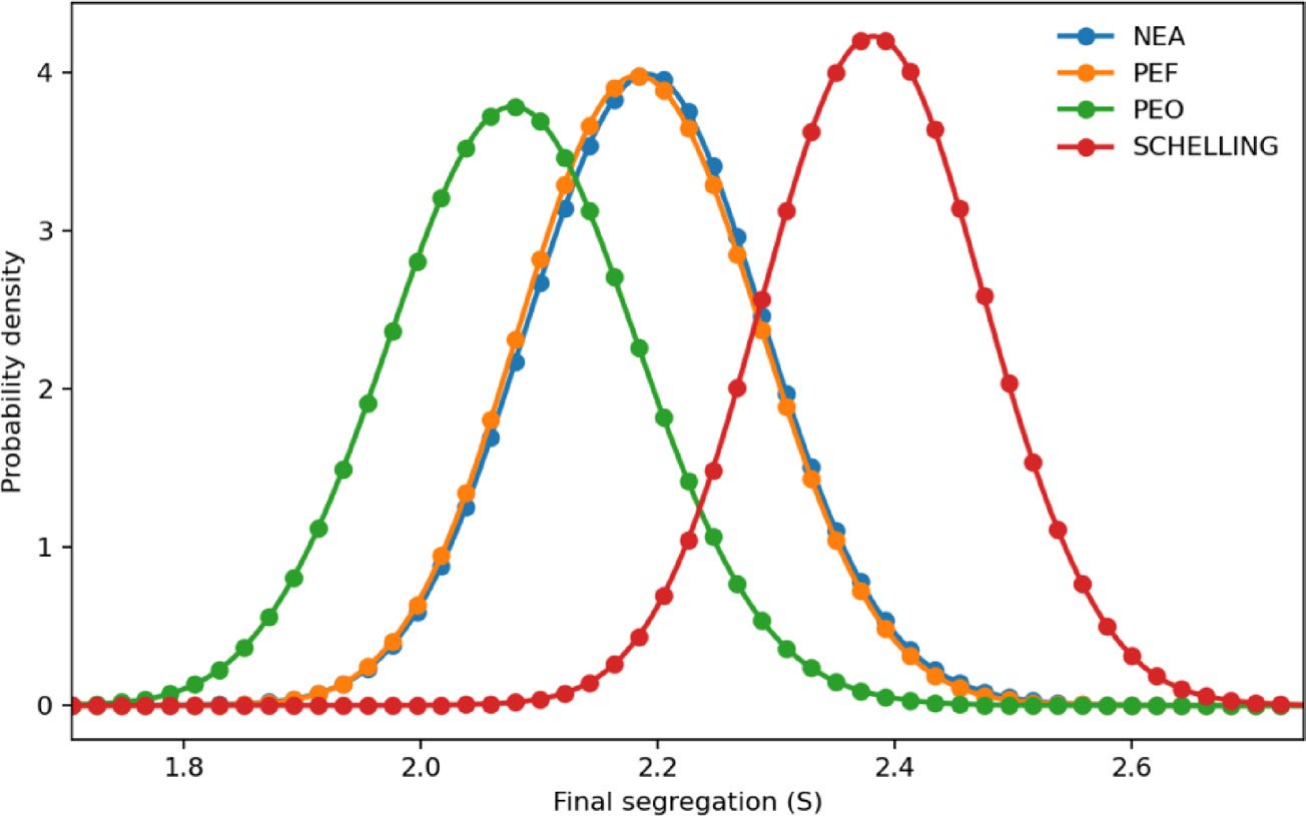
Segregation outcomes across relocation rules. Probability distributions of each strategy. The original Schelling rule produces the highest segregation, while socially oriented rules reduce segregation, with PEO yielding the lowest levels and highlighting the benefit of accounting for both satisfied and unsatisfied neighbors. Lines are smoothed using kernel density estimation (KDE).

We next compare segregation outcomes across relocation rules based on the probability distributions of segregation outcomes (Fig. 1). Each strategy was simulated 10,000 times, with kernel density estimation (KDE) applied for line smoothing. The results show that the original Schelling rule produces the highest level of segregation, whereas PEO yields the lowest. NEA and PEF fall in between, with PEF resulting in slightly lower segregation than NEA, although the difference is marginal. Overall, socially oriented rules substantially reduce segregation relative to the original Schelling rule. The particularly low segregation under PEO underscores the advantage of simultaneously considering both satisfied and unsatisfied neighbors in relocation decisions.

To compare these outcome distributions more formally, we assess *first-order stochastic dominance* (FSD) of segregation outcomes across the behavioral rules. FSD of distribution A over B requires $\:{F}_{A}\left(x\right)\le\:{F}_{B}\left(x\right)$ for all $\:x$, with strict inequality for at least one $\:x$, where $\:F\left(x\right)$ denotes the cumulative distribution function (CDF). For this assessment, we employed two combined methods. First, we conducted two-sample Kolmogorov–Smirnov (K–S) tests, nonparametric tests that determine whether two independent samples are drawn from the same underlying continuous distribution. Second, when the K–S test indicated that two distributions differ significantly, we compared their empirical CDFs to identify which distribution yields higher segregation outcomes and by how much. All K–S tests rejected the null hypothesis at $\:p<0.001$, indicating that all probability distributions originate from distinct underlying distributions. The empirical CDFs revealed the following pattern. First, the original Schelling rule stochastically dominates PEF, indicating markedly higher segregation. Second, NEA tends to exceed PEF in segregation outcomes, though their CDFs intersect slightly near the upper tail, indicating no strict dominance. Third, PEF dominates PEO, and NEA also dominates PEO. Taken together, this pattern implies a monotonic decrease in segregation—from the original Schelling rule to NEA, to PEF, and finally to PEO (Schelling $\:\gg\:$ NEA $\:\ge\:$ PEF $\:\gg\:$ PEO)—confirming the above conclusion.

While there is no absolute benchmark for judging the magnitude of change in the segregation index, all probability distributions under the socially oriented rules in Fig. 1 exhibit clear first-order stochastic dominance by the original Schelling rule. This means that, at any given level of segregation, the Schelling rule produces statistically significantly higher segregation outcomes—and therefore poorer integration—than the socially oriented rules.

**Fig. 2:**
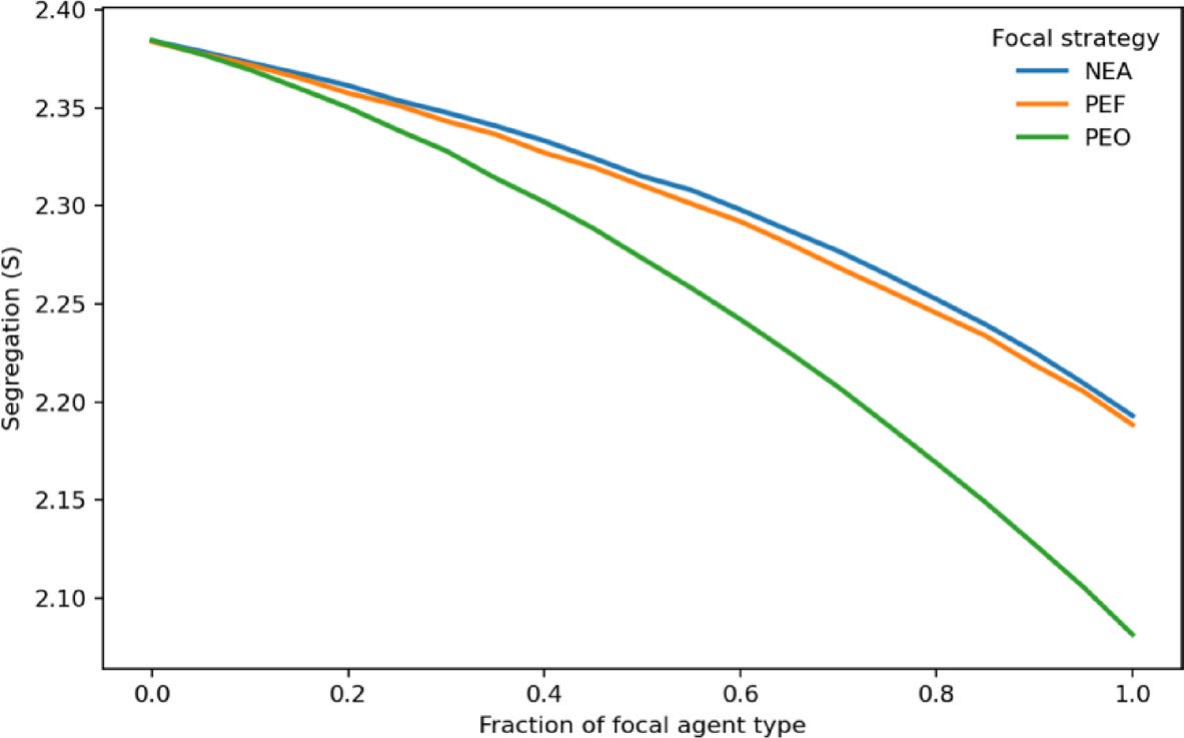
Segregation as a function of the fraction of socially oriented agents. The fraction of agents following socially oriented rules ranges from 0 to 1, with the remainder following the original Schelling rule. Each line represents the resulting level of segregation as a function of the proportion of agents following a given rule relative to the original Schelling rule. Equilibrium satisfaction is reached in all cases. Segregation decreases steadily as the share of socially oriented agents increases, indicating that even partial adoption of socially oriented relocation improves integration. Shaded regions represent 95% confidence intervals but are too narrow to be distinguished from the lines.

Next, we examine how segregation outcomes vary with the fraction of socially oriented agents in the population (Fig. 2). The share of agents following each of the three behavioral rules ranges from 0 to 1, with the remainder adhering to the original Schelling rule. In other words, each line in Fig. 2 depicts the resulting level of segregation as a function of the proportion of agents following a given rule relative to the original Schelling rule. (Final segregation indices across baseline conditions are reported in Table 1). Fig. 2 shows that, consistent with expectations, segregation decreases steadily as the fraction of socially oriented agents increases, yielding more integrated equilibria across replications. Among the tested rules, PEO is the most effective at reducing segregation. While NEA and PEF yield similar outcomes, PEF holds a slight but statistically significant advantage over NEA. Overall, the results indicate that even when only a fraction of agents adopt these rules, the system becomes more integrated, demonstrating that partial incorporation of social orientation is sufficient to improve macro-level outcomes.

**Fig. 3:**
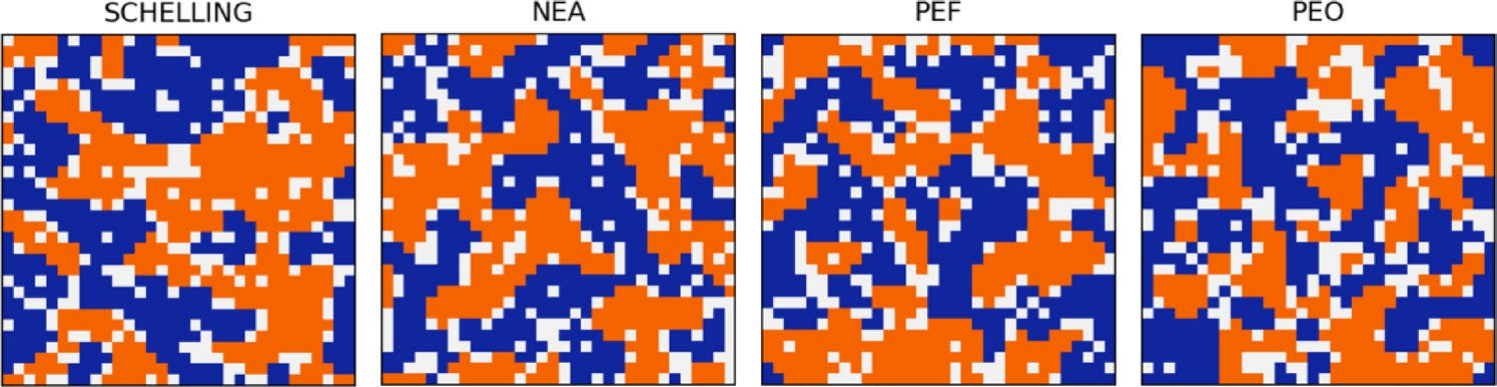
Representative grids at equilibrium. Initial configuration and final grids under each relocation rule. To identify representative outcomes, 10,000 simulations were conducted, and for each condition the run with final segregation closest to the mean was selected. Under Schelling’s original rule, the orange cluster forms a large continuous area near the center, indicating strong segregation. In contrast, under the socially oriented rules—particularly PEO—the same-color regions become smaller and more intermixed with blue, reflecting lower segregation. Consistent with Fig. 1, socially oriented rules yield visibly lower segregation than the original Schelling rule.

Figure 3 illustrates representative simulation snapshots, showing the final grids at convergence. To identify typical outcomes, we conducted 10,000 simulations and averaged the segregation values at equilibrium. For each rule, we then selected the run with a final segregation value closest to this average, ensuring that the displayed grids reflect characteristic results. Consistent with Fig. 1 the socially oriented rules yield visibly lower segregation than the original Schelling rule.

Taken together, these findings demonstrate that social orientation systematically reduces segregation while preserving complete satisfaction, offering a simple yet powerful behavioral refinement that aligns individual mobility decisions with collective welfare and social stability.

### Equilibrium dynamics

**Fig. 4:**
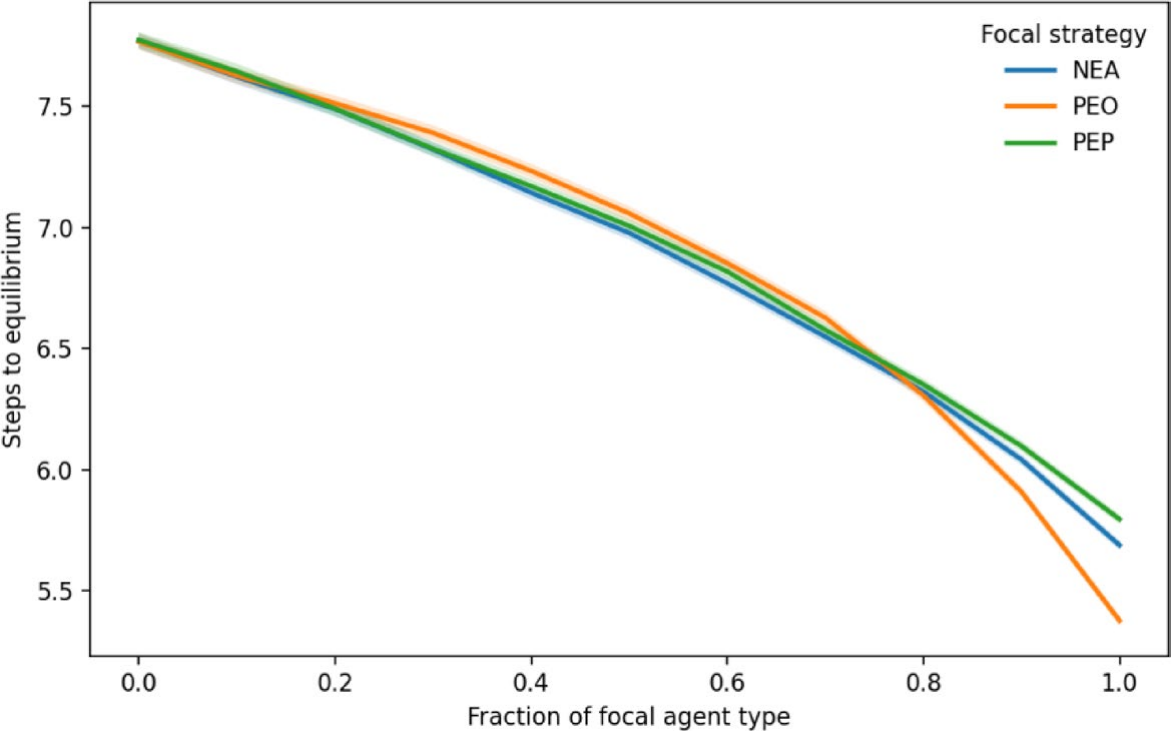
Convergence to equilibrium under socially oriented relocation rules. The fraction of agents following socially oriented rules ranges from 0 to 1, with the remainder following the original Schelling rule. The number of simulation steps required for convergence decreases as the fraction of socially oriented agents increases. Up to a fraction of 0.8, the three rules (NEA, PEF, and PEO) exhibit similar dynamics. Beyond this point, convergence accelerates fastest under PEO, followed by NEA and then PEF. Shaded regions represent 95% confidence intervals but are too narrow to be distinguished from the lines in some areas. Overall, the results indicate that socially oriented relocation not only promotes more integrated equilibria but also accelerates convergence toward them, with implications for social cohesion and resilience.

Finally, we examine how social orientation affects convergence to equilibrium, or global stability. The speed of convergence is relevant for both socially oriented agents and society as a whole for two reasons. First, once achieved, global stability fosters and reinforces mutually beneficial social capital at both the individual and societal levels. Second, it reflects the resilience of the system to exogenous shocks. Figure 4 plots the number of simulation steps required for convergence as a function of the fraction of socially oriented agents. Under all three socially oriented rules, the number of steps decreases as the proportion of socially oriented agents increases. The rules are largely indistinguishable up to a fraction of 0.8, after which convergence accelerates most under PEO, followed by NEA and then PEF. This ordering is slightly different from the segregation results in Fig. 1. Overall, the results suggest that socially oriented behavior not only promotes more integrated equilibria but also accelerates convergence toward them. This finding suggests potential implications for social planners, as a faster attainment of stability might help strengthen social cohesion–and thus social capital–and lower social costs.

**Fig. 5:**
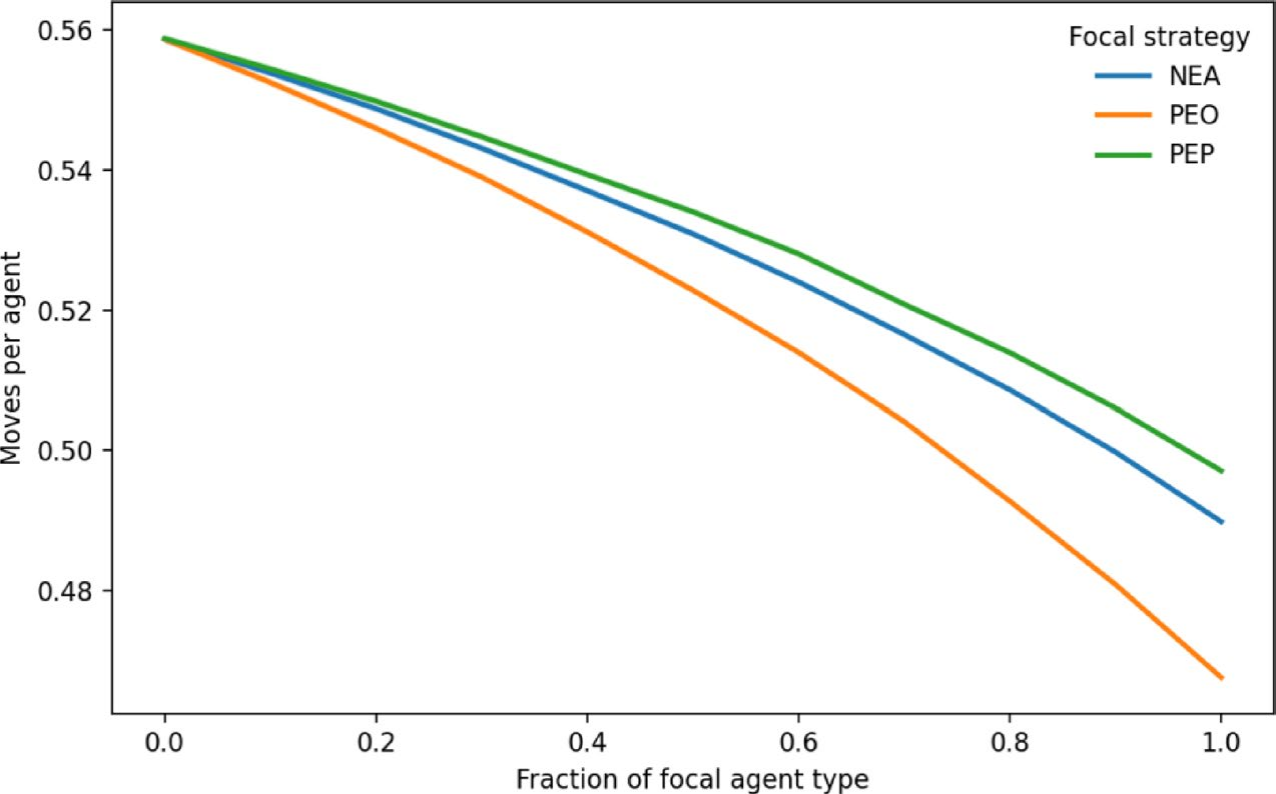
Relocation moves per agent required for equilibrium. The fraction of agents following socially oriented rules ranges from 0 to 1, with the remainder following the original Schelling rule. The figure shows the average number of relocation moves per agent required for the system to stabilize. Shaded regions represent 95% confidence intervals but are too narrow to be distinguished from the lines. Results indicate that the number of moves per agent decreases with the fraction of socially oriented agents across all rules. Among them, PEO requires the fewest moves, followed by NEA and then PEF. These findings suggest that, in addition to promoting integration and stability, socially oriented relocation can reduce the social, psychological, and economic costs of unnecessary moves when moving entails nontrivial costs.

Figure 5 reports the number of relocation moves per agent required for the system to stabilize. (Because the total number of moves naturally declines as the vacancy ratio increases, moves per agent provide a more meaningful measure when the vacancy ratio varies.) The results show that the number of moves per agent decreases with the fraction of socially oriented agents under all rules. Among them, PEO requires the fewest relocation steps per agent, followed by NEA and then PEF. While earlier results suggest that social integration enhances both integration and system-wide stability, these findings further imply that social-oriented behavior also can reduce the considerable social costs of unnecessary moves–whether psychological, social, or economic–when moving entails nontrivial costs.

Together, these results demonstrate that social orientation reduces segregation, accelerates convergence to equilibrium, and lowers relocation moves per agent, thereby reducing social costs and promoting both welfare and stability.

### Robustness checks

**Fig. 6:**
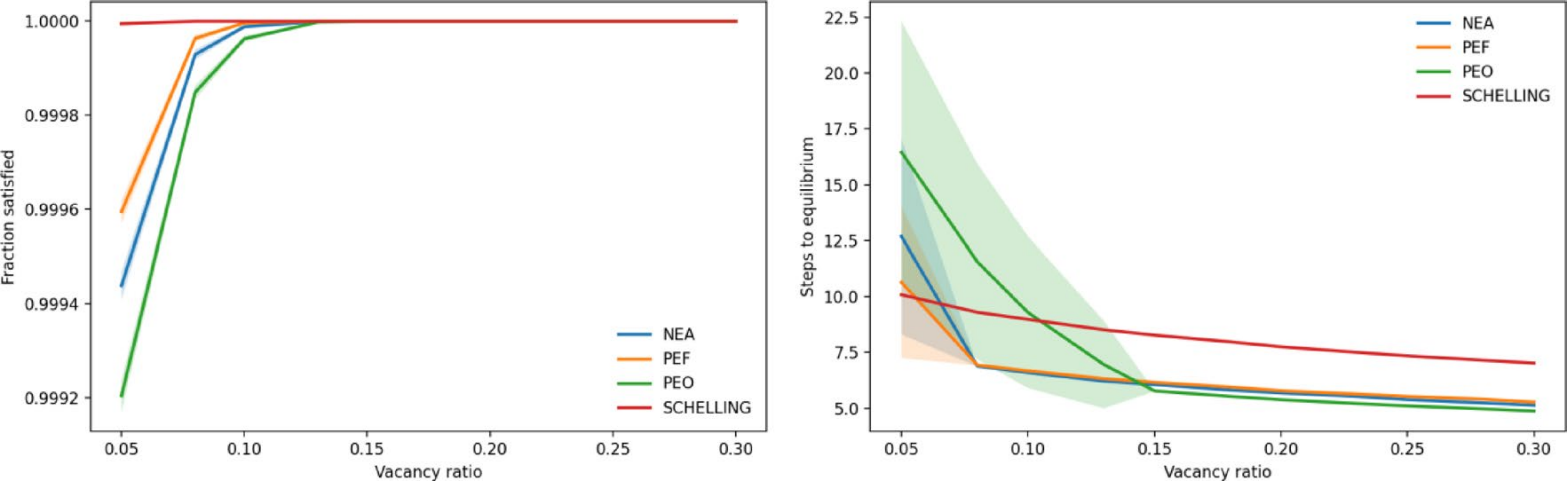
Robustness checks against vacancy ratios. (**a**) Proportion of satisfied agents at equilibrium across vacancy ratios. At very low vacancy ratios (≤ 0.10), complete satisfaction is not always achieved, although the proportion of unsatisfied agents remains below 0.001. (**b**) Steps to equilibrium across vacancy ratios. At low vacancy ratios, socially oriented rules require more steps than the original Schelling rule (notably NEA and PEF at 0.05, and PEO up to 0.125). At higher vacancy ratios, however, socially oriented rules converge more quickly, consistent with the main results. The shaded areas around each line represent 95% confidence intervals.

To assess the robustness of our findings, we conducted additional simulations varying vacancy ratios (0.05–0.30), grid size (32 × 32 to 100 × 100), and neighborhood type (Von Neumann). At low vacancy ratios (≤ 0.10), some inconsistencies emerged: complete satisfaction was not always achieved, although the proportion of unsatisfied agents remained marginal (below 0.001) (Fig. 6a). Moreover, the number of steps to equilibrium was higher under socially oriented rules than under the original Schelling rule—for NEA and PEF at a vacancy ratio of 0.05, and for PEO up to 0.125 (Fig. 6b). These results indicate that socially oriented rules require an adequate number of vacant sites to operate effectively, thereby defining a boundary condition for their applicability. Nonetheless, across all other parameter ranges of grid size and neighborhood type, the qualitative patterns remain unchanged: socially oriented relocation consistently reduces segregation, maintains complete satisfaction, and accelerates convergence relative to the original Schelling rule.

We also conducted additional simulations relaxing the assumption of full environmental observability, under which agents can identify all vacant sites. Even when the set of observable vacancies was constrained to 10, 30, or 50 randomly selected sites, the overall result pattern remained unchanged. Taken together, these findings affirm the robustness of our results.

### Summary of results

Across all baseline simulations, social orientation consistently reshaped the dynamics of Schelling’s segregation model. Compared with the original Schelling relocation, socially oriented relocation produced lower segregation without hampering full satisfaction and faster convergence to equilibrium (i.e., global stability). These effects were robust across replications and parameter settings except lower vacancy ratios, underscoring that a simple behavioral refinement—agents avoiding moves that might destabilize others—can transform macro-level outcomes. Together, the results highlight social orientation as a powerful microfoundation linking individual decision-making to collective outcomes. Specifically, our findings show that incorporating social orientation into Schelling’s framework not only reduces segregation but also accelerates stabilization, offering a simple yet powerful behavioral refinement that lowers social costs and fosters more resilient, integrated communities. These findings motivate the broader theoretical and empirical implications we discuss in what follows.

## Discussion

Our results show that introducing social orientation into Schelling’s segregation model reshapes its macro-level dynamics. Unlike the original rule, which reproduces considerable segregation, socially oriented relocation reduces segregation, maintains satisfaction, and accelerates the attainment of global stability. These outcomes demonstrate how locally rational behaviors that preserve social capital by considering others’ satisfaction can scale into welfare-enhancing equilibria that also produce more integrated and resilient communities. From a broader perspective, our research also connects to work in coevolutionary dynamics that highlights similar adaptive feedback mechanisms^56^. Although our model does not include institutional adaptation, it follows a comparable feedback process in which socially oriented relocation reshapes the neighborhood environment that, in turn, influences subsequent decisions. This iterative dynamic—where micro-level adjustments generate macro-level configurations that feed back into individual satisfaction—parallels the adaptive coupling between behavior and environment emphasized in coevolutionary models^56^.

### Theoretical implications

 Our findings extend Schelling’s original insight that simple local rules can produce complex social outcomes. In contrast to the destabilizing dynamics of Schelling’s other-ignorant relocation rule, social orientation establishes conditions under which individual rationality, collective welfare, and social integration are mutually aligned. This provides a stronger microfoundation for segregation models by showing how boundedly rational, socially sensitive agents can generate more integrated equilibria. More broadly, our results contribute to theories of self-organization and emergent order by demonstrating how realism-based local decision-making heuristics can reshape global outcomes.

### Applications to empirical contexts

 These insights are relevant across multiple domains. In urban settings, residents naturally avoid neighborhoods prone to instability, and our results suggest that socially oriented preferences can counteract destabilizing forces such as segregation or rapid gentrification by fostering more resilient communities. In organizational contexts, similar dynamics apply to team formation and group stability: members who avoid moves likely to unsettle colleagues can strengthen cohesion and reduce turnover. Taken together, these parallels illustrate how social orientation provides a unifying principle across domains where individual mobility decisions aggregate into collective outcomes.

### Suggestions for policy makers and social planners

 Our findings suggest several avenues for fostering stability and integration. Policy makers can encourage such outcomes by improving transparency (e.g., publishing neighborhood stability indicators), designing incentives that reward community-preserving moves, and providing institutional support such as vacancy buffers or coordinated relocation programs. Social norms and community engagement can further reinforce stability by embedding social orientation as a shared value. Taken together, these measures align individual mobility choices with broader societal outcomes such as reduced segregation, collective welfare, and stability, without requiring information-intensive taxation mechanisms^36,37^. This alignment, in turn, lowers social costs and fosters more resilient and integrated communities.

### Limitations and future directions

 Our study abstracts from several real-world complexities that warrant future work. First, our model abstracts from individual heterogeneity in satisfaction thresholds and social orientation propensities. Future research could relax this simplifying assumption to examine how variations in these parameters influence collective dynamics and segregation outcomes. Second, while the model assumes two-step observability, the actual perception of neighborhood stability likely varies across individuals and contexts, reflecting differences in cognitive capacity, attention, and social awareness^57,58^. Third, we assume simple greedy search behavior, though more sophisticated heuristics could further influence dynamics. Future research could also integrate network structures beyond grids, such as small-world or scale-free networks, to examine how social orientation operates under different topologies^59,60^. Fourth, empirical validation using residential mobility data or organizational turnover patterns would be a critical step in testing the model’s applicability. Fifth, following Schelling’s original formulation, our model allows only dissatisfied agents to relocate to sites that meet their satisfaction threshold. While this satisficing rule preserves the model’s parsimony and behavioral clarity, it excludes better-reply or incremental-improvement moves that could generate richer dynamics—an extension we identify as a promising direction for future research. Finally, the implications of our model should be interpreted with caution, as real urban segregation processes involve additional factors—such as housing prices, urban amenities, and labor market dynamics—that fall outside the scope of this simplified framework. Future research could extend the model to incorporate these structural and economic dimensions, providing a more comprehensive understanding of segregation dynamics.

## Conclusion

By extending Schelling’s segregation model with social orientation, we show that individual-level tendencies to preserve social capital and community can reduce segregation and facilitate the attainment of global stability. These results provide a stronger behavioral foundation for models of segregation and highlight how social orientation, combined with bounded rationality under local observability, shapes collective patterns. Beyond theoretical insight, the findings suggest that fostering socially oriented behaviors—whether in urban mobility or organizational settings—may promote more integrated, resilient, and sustainable communities.

## Data Availability

All simulation codes developed and used in this study are publicly available to enable replication, validation, and extension of the results at [https://github.com/kyuhojin/schelling-social-orientation.git](https:/github.com/kyuhojin/schelling-social-orientation.git) .
